# Beehive Air Sampling and Sensing Device Operation in Apicultural Applications—Methodological and Technical Aspects

**DOI:** 10.3390/s21124019

**Published:** 2021-06-10

**Authors:** Andrzej Szczurek, Monika Maciejewska

**Affiliations:** Faculty of Environmental Engineering, Wroclaw University of Science and Technology, Wybrzeże Wyspiańskiego 27, 50-370 Wrocław, Poland; andrzej.szczurek@pwr.edu.pl

**Keywords:** gas sensor, bee, monitoring, indoor air quality, electronic nose

## Abstract

The basis of effective beekeeping is the information about the state of the bee colony. A rich source of respective information is beehive air. This source may be explored by applying gas sensing. It allows for classifying bee colony states based on beehive air measurements. In this work, we discussed the essential aspects of beehive air sampling and sensing device operation in apicultural applications. They are the sampling method (diffusive vs. dynamic, temporal aspects), sampling system (sample probe, sampling point selection, sample conditioning unit and sample delivery system) and device operation mode (‘*exposure-cleaning*’ operation). It was demonstrated how factors associated with the beehive, bee colony and ambient environment define prerequisites for these elements of the measuring instrument. These requirements have to be respected in order to assure high accuracy of measurement and high-quality information. The presented results are primarily based on the field measurement study performed in summer 2020, in three apiaries, in various meteorological conditions. Two exemplars of a prototype gas sensing device were used. These sensor devices were constructed according to our original concept.

## 1. Introduction

Honey bees are critically important for the environment and to the economy [1]. Recently, the population of these insects has been disappearing at an alarming rate throughout the world. This dangerous phenomenon is still poorly recognized and understood. Probably, it is caused by the combined effect of many interrelated, difficult, or impossible to control factors, e.g., shifting flowering seasons due to climate change, reduced floral diversity, use of pesticides, habitat loss, lack of genetic diversity, insect parasites and harmful microorganisms [2]. To prevent the losses of bee colonies, populations of these insects have to be maintained in an optimal state of health and afforded opportunities to grow.

Good beekeeping practices need solid, comprehensive information about the state of honey bee colonies, the beehive environment as well as events that may require the beekeeper’s action [3]. Traditionally, it comes from observation of honeybee behavior as well as conditions associated with beehives and the environment. This approach may be widely applied; however, it is time consuming and requires great experience. In addition, the obtained information is subjective since it depends on the individual abilities of apiary inspectors or beekeepers.

The disadvantages of visual inspection mean that monitoring based on instrumental methods raises great interest in apiculture [4]. This strategy is focused on measurements of parameters such as temperature, air humidity, analysis of sound and vibration of a beehive, counting outgoing and incoming bees (forager traffic) as well as the number of bees in the hive entrance area, video observation, weighing of the colony, and determination of the content of beehive air [5,6,7,8,9]. The obtained data may provide information about the health, productivity, and behavior of honey bees, events and factors influencing the state of bee colony, and physical and chemical conditions inside beehives. Continuous monitoring based on measurements of physical and chemical parameters has been proposed to study colony growth, activity, metabolic processes, genetics, and behavior. In this way, beekeepers can be informed about swarming/pre-swarming state, extreme nectar flow, queenless state, broodless state, dead colony, starving, and first cleaning flight in spring, as well as diseases, including colony collapse disorder, to evaluate treatment effects. The use of instrumental measuring methods allows to obtain information without invasive hive inspections and disrupting the beehive environment.

The aim of this paper is to discuss the selected issues regarding the application of gas sensing devices in beekeeping. We shall focus on beehive air sampling and the operation of the measuring instrument, which is subordinate to the sampling method. Our attention was directed to the impact caused by the environmental factors (beehive and ambient environment) because during gas sensing device operation in field conditions, they significantly affect the measuring process and make it more complicated. Practical aspects and technical limitations are presented. Sharing the observations resulting from the experience acquired during many measurement sessions performed in the field, in different apiaries, owned by different beekeepers and in various ambient conditions, is the principal value of this work.

The structure of the paper is as follows. In Section 2, the issue of beehive air measurements for beekeeping is addressed. We note that the gas sensing application in this domain is promising. Section 3 presents the scope of field studies, which have been the source of the most important observations we shared in the paper; the prototype gas sensing device is introduced. Section 4 is dedicated to the presentation and discussion of the obtained results. It concentrates on two issues. They are various aspects of: (1) the sampling method and sensing device operation (Section 4.1), and (2) the sampling system itself (Section 4.2). Both are examined in view of performing beehive air measurements in real conditions. Conclusions are presented in Section 5.

## 2. Beehive Air Measurement for Beekeeping

It has been noticed that the valuable information for the modern apiculture is associated with the chemical composition of beehive air. The gaseous mixture inside the hive is a unique and complex mixture of many different volatile compounds. These substances are produced and emitted by bees themselves (e.g., pheromones, other chemicals released to repel pests and predators, metabolites, etc.); honey, nectar, larvae, beeswax, pollen, and propolis; materials out of which hives are constructed (wood, paint, plastic, etc.) and outside sources, e.g., vehicles, farms, industries, and households in the vicinity of hives. The specific chemical composition of beehive air is essential for the existence of a honey bee colony [10,11]. It creates the odor of the nest, which has the primary and vital function of determining the identity of the colony members. Some volatile compounds drive honeybees’ hygienic behavior. The chemical composition of beehive air reflects sanitary conditions inside the hive and the health condition of the honey bee colony [12].

Today, there are a number of analytical methods and instruments, e.g., infrared analyzers, Fourier-transform infrared spectrometers (FT-IR spectrometers), ion mobility spectrometers (IMS) or gas chromatographs with an appropriate detector (usually FID, tuned mass spectrometer MS or other mass-selective detector), which are capable of detecting, identifying, and quantitating the specific chemical species in beehive air [13]. They offer objectivity, good accuracy, sensitivity, and repeatability. However, the cost of such analytical instruments is prohibitive and beyond the reach of the average beekeeper. This equipment is bulky in size, heavy in weight, inconvenient to transport, and its energy consumption is high. The measurement procedure is time-consuming and labor-intensive. Additionally, trained and experienced personnel is required to attain reliable measurement results. Analytical instruments are still mainly dedicated to laboratory works when the speciation is absolutely necessary.

The alternative for the conventional equipment, to a certain extent, are measuring instruments based on a sensor technology [14]. They allow to reduce the cost of tests and increase the number of applications in field conditions. Low-cost sensing systems often cost orders of magnitude less than conventional instruments. Sensor devices can be used in modern apiculture for: (1) the identification and quantification of chemical substances that are indicators characterizing the state of the honey bee colony as well as conditions inside beehives, and (2) the classification of beehive air quality [15]. The second variant is more promising. It results from the properties of beehive air indicators. These substances occur in very complex gaseous mixtures. Their concentration is very low, at the level of ppb or ppm. The detection limit of commercial gas sensors usually does not allow to detect these substances. Majority of gas sensors are not selective enough to identify a single chemical species in the gaseous mixture. Additionally, indicators describing honey bee colony states are poorly understood and recognized. Therefore, a holistic approach based on the classification of beehive air quality is frequently proposed and taken into account in the potential applications of an equipment based on gas sensors.

Sensing devices, designed for the classification of complex gaseous mixtures, are very often called electronic noses [16]. The working principle of these instruments is inspired by the sensing mechanism of mammal senses. A typical e-nose device includes: a sampling system, an array of chemical gas sensors, a signal conditioning and data acquisition module, and a computer microprocessor with sample classification method (pattern-recognition algorithm). Gas sensors incorporated into the e-nose respond to a wide spectrum of volatile compounds. Hence, the multi-sensor array produces combined output pattern in response to many substances present in the sample mixture. The collective analysis of data originating from the sensor array is the basis for the classification of the tested gas mixtures. The information is obtained by comparing the output from the sensor array to reference databases, produced by the instrument training that is based on mathematical and statistical processes involving pattern recognition algorithms. Classification algorithms use chemometric methods dedicated for processing multivariate data. The electronic nose technology provides effective solutions for the accurate, reliable, and speedy identification of multi-component gas mixtures. It was shown that measuring abilities of the instruments based on gas sensors and pattern recognition methods allow to detect varroosis and determine the infestation rate in individual beehives [17,18]. Despite the increasing progress in sensor technology, gas sensing devices of this kind remain largely limited by environmental and technological factors [19,20,21,22]. The practical application of these devices is still challenging, and several problems have to be solved.

A number of studies have shown that proper gas sampling and operation has a strong influence on the measurement characteristics of the gas sensing device [23,24]. Sampling is one of the most important stages of a measurement process. The sample is the actual source of information about the studied object. Therefore, its proper acquisition is especially important to ensure the information accuracy. If a sample is not collected properly, the whole measurement process is erroneous. Even perfect sensors cannot remove errors generated by faulty sampling. Proper operation of a measurement instrument is essential to ensure that high-quality information is obtained based on the gas sample.

## 3. Experimental Part

### 3.1. Gas Sensing Device

The gas sensing devices used in our work were direct reading instruments. They provide single values of responses per sensor, at a given point in time, that allow to classify different complex gas mixtures. These devices were developed in the Laboratory of Sensor Technique and Indoor Air Quality Studies, WUST. The instruments were dedicated not only for professionals. They were easy to use and worked with the minimal operator attention. The measurement process was automated. They allowed to perform both short- and long-term measurements. The data was provided in real time. The embedded GSM-module allowed for the remote internet connection and the collection of the measurement results. The construction of these devices made them well-suited to work in field conditions. The sensing devices were simple to deploy in apiaries in remote localizations. They allowed to perform online measurements, directly at the beehives. There was not required sample transport to the laboratory or its special handling to avoid deterioration of the collected gas. The serious limitation of the devices was high energy consumption. The power consumption of the measurement device should not exceed 40 W and typically it is around 10 W (without heated gas line). Sensor heaters consume around 3 W. The temperature control of the sensor chamber requires about 3 W. The electronics consume around 1 to 1.3 W. Data transmission requires less than 1 W. The pump needs about 1.7 W. The heated gas line consumes 7 W. In order to assure 10 h of continuous operation, we used high amp-hour batteries REC22-12-(Capacity 22Ah @ 20hr-rate, Voltage 12 V) which were heavy (weight approximately 6.2 kg) and expensive. Independent power sources, such as solar panels, offered an opportunity to work in localizations, where the electrical supply network was not available. However, beekeepers did not accept this solution. The gas sensing devices developed in our lab can be used as portable as well as stationary instruments. Currently, a handheld version is not yet available. However, compared to traditional analytical instruments based on gas analysis methods, such as gas chromatography mass spectrometry (GC-MS) and Fourier transform infrared (FT-IR) spectrometry, gas sensor devices have the potential to be small, compact, fast, inexpensive and cost-effective in operation.

**Figure 1:**
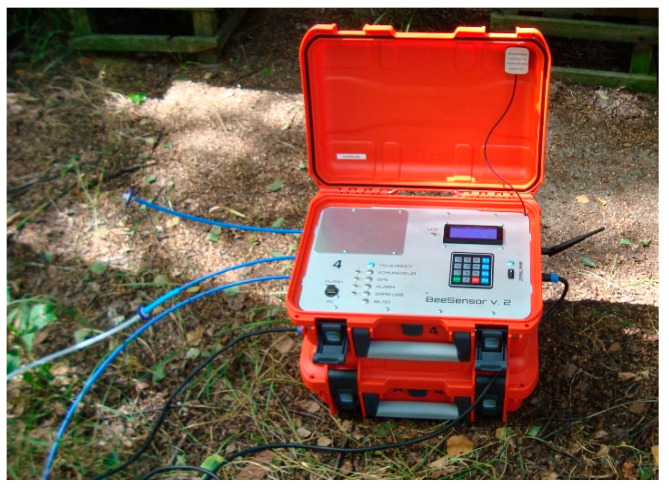
Gas sensing device which was used in our experiments.

The gas sensing device which was used in our experiments is shown in Figure 1. It consisted of the following parts:

- gas sampling unit with sample delivery system;
- array of six low-cost semiconductor gas sensors (TGS822, TGS823, TGS826, TGS2600, TGS2602, TGS2603) with partial or overlapping sensitivities;
- circuit for an analog signal conditioning;
- module for data acquisition, and storage;
- signal preprocessing module;
- pattern recognition machine;
- GSM module for data transmission;
- GPS module for instrument localization.

As shown in Figure 1, the gas sensing device is portable and protected by two independent waterproof cases. The top case hosts the measuring instrument. The bottom case contains batteries. There is a cable connection between the two parts. The device is fitted with two gas inlets: ambient air inlet for regeneration gas preparation (first from the top), gas sample inlet (second from the top) and gas outlet. Keypad allows for the direct communication with the instrument. Signaling diodes provide the information about its status.

The major element of the measurement device was the gas sensor array. It was composed of commercially available semiconductor gas sensors, which are offered by Figaro Engineering, Osaka, Japan [25]. We chose the following ones: TGS822, TGS823, TGS826, TGS2600, TGS2602 and TGS2603. According to the producer, for sensors included in the array, the target gases are as follows: TGS822—organic solvent vapors, TGS823—organic solvent vapors, TGS826—ammonia, TGS2600—air contaminants (high sensitivity to hydrogen and carbon monoxide), TGS2602—air contaminants (high sensitivity to odorous gases such as ammonia and H_2_S) and TGS2603—air contaminants (high sensitivity to odorous gases such as amine-series and causing sulphurous odour). However, the individual semiconductor gas sensors are sensitive to multiple volatile compounds and their sensitivity ranges are partially overlapping. On purpose, sensors applied in our device had different detection ranges. In particular, the detection range of sensors TGS2600, TGS2602 and TGS2603 is from 1 ppm to several dozen ppm. The detection ranges of sensors TGS822, TGS823 and TGS826 are from 10 ppm to more than 1000 ppm. The selection of sensors was based on the assumption that: (1) we should concentrate on volatile organic compounds as the source of information about bee colony, (2) the composition of beehive air is complex, largely unknown, and highly variable.

The gas sensor signal was defined as the voltage across the voltage divider, which consists of the chemosensing element and the load resistor. The method to convert the sensor resistance R_S_ to the recorded signal, V_RL_, is given by Equation (1).

V_RL_ = V_C_ R_L_/ (R_L_ + R_S_) (1)

where: V_C_ is the circuit voltage equal to 3.3 V, R_L_ is the value of the load resistor equal to 10 kΩ for TGS sensors series 800 and 2 kΩ for TGS sensors series 2000.

This paper is focused on the first element of the gas sensing instrument, which is the gas sampling and sample delivery system and the device operation mode. Two exemplars of the gas sensing device were used for testing these elements of the equipment in real field conditions.

### 3.2. Apiaries

**Figure 2:**
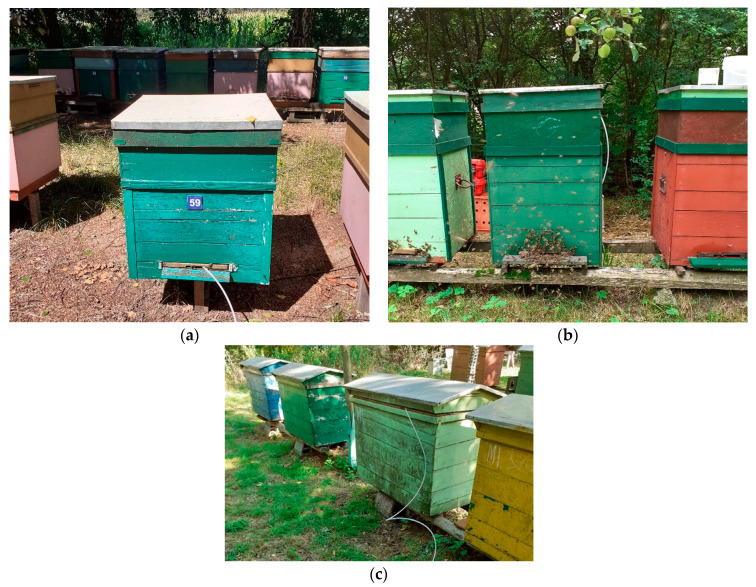
Different kinds of beehives, which were included in the study. They were wooden. They were built by beekeepers. (**a**) Apiary 1 located on the border between forest and field, (**b**) Apiary 2 located in the garden, (**c**) Apiary 3 located on the border between forest and field.

The presented results are primarily based on field studies. The field studies were performed in July, August, and September 2020. The measurements were carried out in three professional, private apiaries, located in southwestern Poland, in dolnośląskie and opolskie voivodships. Two apiaries were located on the border between forests and fields. One was located in the garden. We encountered various kinds of beehives in the apiaries. They are displayed in Figure 2. The majority of beehives were wooden ones. They were built by beekeepers themselves. The beehives were the modified versions of some standard constructions, or they were beekeepers’ own constructions. The adjustment of well-established constructions to the local, specific needs is a common situation among Polish beekeepers.

The measurements were performed during the daytime, in various meteorological conditions. The weather was mild or hot with temperatures ranging from 20 °C to above 30 °C in the middle of the day. Most days were sunny. Single days were cloudy. Rainy weather was encountered only once.

## 4. Results and Discussion

In our study, gas sampling was the operation of removing a part of beehive air (of proper volume) from the whole beehive in such a manner that the sample represented, within measurable limits of error, the chemical composition of this gas. The objective of sampling was to ensure that the sampled gas was representative. The representative gas sample has the same characteristics as beehive air at the sampling point and time.

Our studies have shown that several issues have to be taken into account in the development of the beehive air sampling method and equipment. The most important are: properties of beehive air, environmental conditions, honey bee behavior, construction and materials of sampling equipment, and characteristics of gas sensors. Other factors such as convenience, site accessibility, technical limitations and costs also play an important role in developing the sampling strategy. In all test situations, consideration must be given to: fluctuations of pressure, temperature, or concentration of beehive air components due to uncontrollable variations in the beehive and outdoor environment moisture content and expected interferences.

### 4.1. Sampling Method and Device Operation

#### 4.1.1. Diffusive Sampling

Online measurements of beehive air can be performed using diffusive or dynamic methods of gas sampling. The operating principle of the diffusive sampling relies on the movement of a sample due to the concentration gradient established between the tested gas and a sensor. The gas is taken from the beehive at a rate controlled only by a physical process such as diffusion through a static air layer or a porous material and/or permeation through a membrane. The gas transport is described by Fick’s First Law. Main advantages of the diffusive sampling are cost effectiveness and simplicity. This method is especially attractive when limitations in dimension and energy consumption are the critical factors. The diffusive sampling does not require a power supply since the active movement of beehive air through the sensor device is not involved. Natural air currents deliver gas sample to sensors. As a pump or fan is not applied to move the gas, the sampling process is completely quiet. This is an important advantage in applications in which the measuring device has to be located inside the beehive. Diffusive sampling also has serious disadvantages. The performance of this method is dependent on the diffusion coefficient, which is affected by the unique chemical and physical properties of gas under test, construction of the sensor housing (its geometry and shape) as well as by a number of environmental factors. Sensor devices based on gas diffusion have to deal with fluctuations of environmental conditions. The dependence of the diffusion coefficient on temperature and pressure significantly reduces the sampling rate when stagnant conditions occur. It is a strong limitation if the measuring system presents the memory effects. The relatively slow gas exchange in diffusive sampling causes the responses of the sensors to be slower. It may be problematic and undesirable in some applications. In our work, we gave preference to dynamic sampling.

#### 4.1.2. Dynamic Sampling

Due to the conditions inside a beehive, a measurement device should be located outside the beehive. The consequence of such a choice is that the dynamic sampling must be involved in the measurement process. In this method, the representative portion of the tested gas is continuously withdrawn from the sampling point and transported, at a controlled flowrate, to the sensor chamber for real-time measurements. The movement of the sample is activated by a pump. The dynamic sampling ensures continuous and constant gas flow across the measurement unit. Dynamic sampling is especially valuable for assessing a temporal variability of beehive air properties. Usually, this gas is highly heterogeneous and its content changes dynamically in time.

#### 4.1.3. Continuous Sampling

Theoretically, continuous sampling coupled with sensor measurements may rapidly provide data regarding changes in a beehive environment. In practice, there are some constraints in the application of this strategy. The shortcomings result, e.g., from the properties of gas sensors—especially chemiresistors. Many of these devices present a drift, memory effect and a tendency to become saturated under the influence of some organic compounds. It leads to poor reversibility and reproducibility of the sensor responses and is reflected in a false interpretation of the measurement results. Among the most serious limitation of many gas sensors is the “long-term” drift problem. It may be identified and characterized by significant temporal variations of sensor signals, changes in the sensing material baseline and measurement sensitivity over time. Sensors with “long-term” drift do not give stable responses over a long period of time, even when they are exposed to identical gas mixtures. Saturation of the sensing material, long term drift, as well as the memory effect originate from numerous, sometimes unrecognized processes, e.g., aging, contamination or poisoning of a sensing material. It is difficult to eliminate causes of these processes. The appropriate operation of sensor device allows to reduce these disadvantages.

#### 4.1.4. ‘Exposure-Cleaning’ Operation

**Figure 3:**
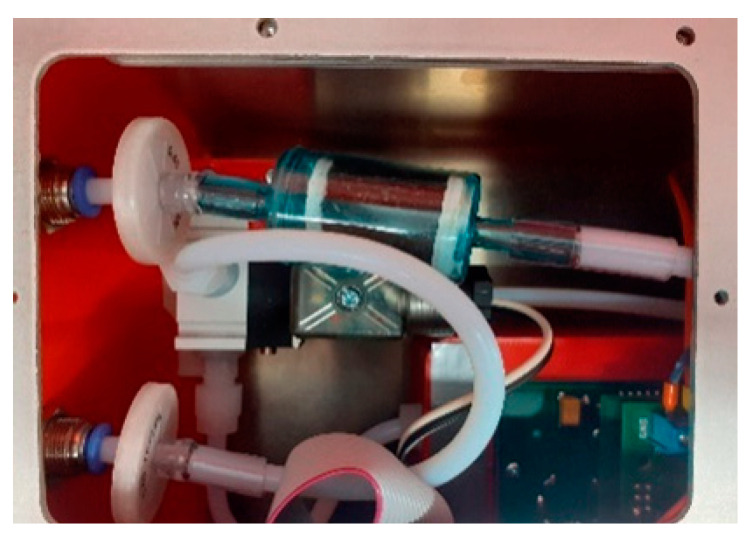
The interior of gas sensing device. Top—the gas line used for delivering the cleaned ambient air for sensors regeneration; Teflon filter and charcoal filter were mounted on this line. Bottom —the gas line used for delivering gas sample; only Teflon filter was mounted on this line.

The hybrid method, which is a combination of exposure and cleaning operations, may be a good alternative for continuous sampling. In such an approach, the portion of the tested gas is taken and transferred to the sensor chamber in a periodic manner. The gas sampling and exposure are performed simultaneously. The exposure of each sensor to the test gas is followed by a cleaning phase, which is aimed to restore the sensor characteristics. This operation is carried out by means of a stream of a reference gas. In our construction, this gas was dry air, purified by a simple filtering of atmospheric air. The filter consisted of an activated charcoal. The respective part of the reference gas line, inside the gas sensor device, is shown in Figure 3. The Teflon filter and the charcoal filter, which were mounted on the gas line used for delivering the cleaned ambient air for sensors regeneration, are displayed. Filtration was used to reduce and stabilize the concentrations of organic compounds in the ambient air, which was delivered to the sensor system. Based on our calculations, the charcoal filter was sufficient to perform measurements for the whole season, i.e., for six months of continuous measurements.

**Figure 4:**
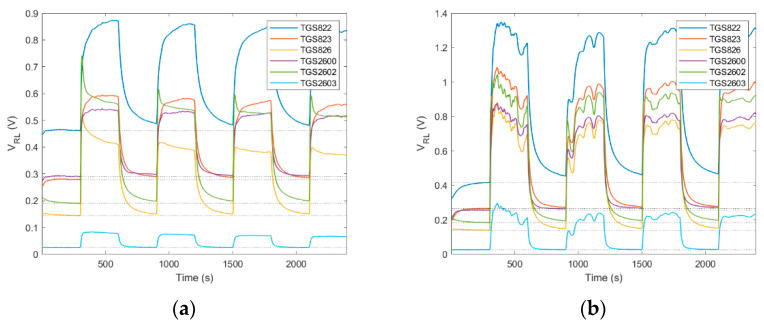
Raw sensor signals resulting from ‘*exposure-cleaning*’ operation repeated four times, preceded by the establishment of the initial baseline; (**a**) signals of most sensor approach the steady state during exposure to beehive air; (**b**) sensor signals do not attain steady state due to rapid fluctuations of the tested gas composition or obstruction of its access to sample probe. Dotted lines show the baseline of the first sensor signal as indicated by the initial exposure to clean air.

Time required for performing the ‘*exposure-cleaning*’ operation is an important factor influencing the application potential of gas sensing devices. In our work, the sampling and exposure time was 5 min. It was determined on the basis of the beehive air measurement data analysis as the total amount of time sufficient to reach 90% of the saturation value by the measurement signals, see Figure 4a. In general, the full stabilization of the semiconductor gas sensors in beehive air measurements cannot be expected since these devices seldom reach an equilibrium state with volatile organic compounds (VOCs) in a short time. Usually, the response time of gas sensors ranges from seconds up to several minutes. It is caused by slow dynamics of the sensing material. Moreover, rapid fluctuations of the tested gas composition, or variation of its access to the sample probe are oftentimes encountered in beehive air measurements, see Figure 4b.

The duration of the cleaning phase results from the time required for achieving full regeneration of the sensing material. In our device, this value was set at the level of 5 min. Based on multiple measurements, this time was sufficient to attain the repeatable level of regeneration of the sensing material, although the regeneration was not always complete. This fact is illustrated in Figure 4, by the sensor baseline shift upon subsequent regenerations as compared with the first exposure to clean air. In general, the required recovery time may be longer than the response time. Oftentimes, it is the factor which limits achieving high measurement frequencies for continuous monitoring applications.

**Figure 5:**
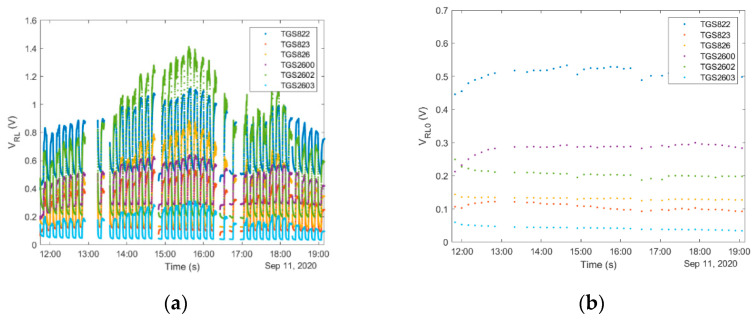

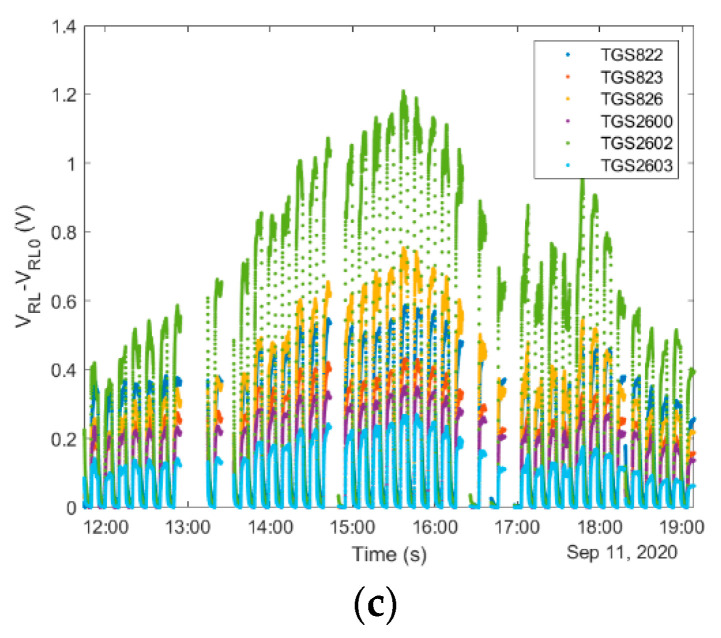
Results of bee colony monitoring using ‘*exposure-cleaning*’ operation. On the measurement day, the beehive air did not display any pattern of temporal variation, which could be associated with the measurement system operation. In particular, sensor signals did not decrease regularly in time; (**a**) raw sensor signals, (**b**) baseline for subsequent ‘*exposure-cleaning*’ operations, and (**c**) sensor signals after differential baseline correction.

**Figure 6:**
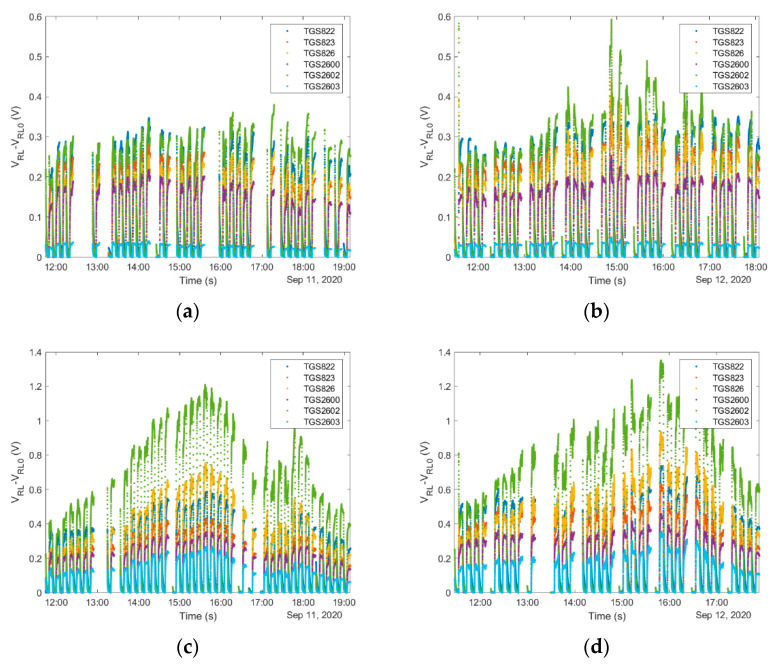
Results of monitoring two bee colonies, living in one apiary, in adjacent beehives during two consecutive days. (**a**) Bee colony 1 on day 1; (**b**) bee colony 1 on day 2; (**c**) bee colony 2 on day 1; (**d**) bee colony 2 on day 2.

Dynamic sampling prevents changes of gas sample composition before it reaches the sensor chamber. The method based on ‘*exposure–cleaning*’ operation ensures the rapid transfer and exchange of gas inside the measurement chamber as well as fast regeneration of sensors. However, a high sampling rate can change the beehive air composition around a sampling probe. It is due to natural ventilation of the beehive. For that reason, the amount of air taken from a beehive should be relatively small, although sufficient to obtain a representative sample. This poses a requirement on the flow rate of the sampled gas. In our system, the gas flow rate was set at the level of 1 L/min. It was not observed that this flow rate applied for a five-minute sampling period caused a measurable change of the signal resulting from the dilution of beehive air in the surrounding of the sampling point. This conclusion was inferred from the observation that sensor signals recorded during multiple subsequent ‘*exposure-cleaning*’ operations were not systematically decreasing, see Figure 5, unless due to changes of beehive atmosphere caused by bee colony activity e.g., its diurnal cycle, see Figure 6c,d.

In the designed sensor system, cleaned ambient air was not only used for sensor regeneration and cleaning of the measuring system; it was also the reference gas. The signal measured at the end of the cleaning phase was settled as the baseline value, V_RL0_, and it was used as the reference for the subsequent exposure to the gas sample. It was assumed that the useful response of sensor array (Figure 5c) to the tested gas should be the difference between the signal measured during the exposure V_RL_ (Figure 5a) and the baseline V_RL0_ (Figure 5b). From the baseline behavior during the day (Figure 5b), it may be noticed that sensor regeneration process was effective. Hence, the information collected during a particular exposure was not biased by the previous exposure.

Based on the experimental data, measurement results obtained from a single ‘*exposure-cleaning*’ operation do not provide accurate information about beehive air. Data analysis has shown that valuable information was obtained if a measurement session consisted of at least four consecutively performed ‘*exposure-cleaning*’ operations, see Figure 4. In other words, measurement sessions should consist of a minimum of four tests. The monitoring program of beehive air may be based on one or many separate measurement sessions, performed in different time periods; see, e.g., Figure 5 and Figure 6.

#### 4.1.5. Consideration of Temporal Factors Influencing Operation of Gas Sensing Devices in Beekeeping

The gas sample should be representative in terms of any temporal variations associated with the monitored process. Hence, ‘*exposure-cleaning*’ operation may have to be performed many times at different time points. The duration of the individual measurement sessions, their number, and the period of time when they are performed depends on the purpose of the monitoring program, the temporal variations of factors influencing beehive air, and also on technical conditions. The selection of the appropriate time for measurements has a principal significance for the realization of beehive monitoring. There is a high probability of obtaining misleading information when the measuring session is performed in an inadequate time period. This problem is mainly associated with the temporal variability of beehive air [26]. The chemical composition of air inside the beehive presents a temporal variability, see Figure 6. It is caused by internal factors, but it is also influenced by the outdoor environment. The concentration of VOCs in the beehive air and their variation in time may be influenced by the outdoor level of these substances. There are many sources of VOCs in the environment, both biotic and abiotic, with the time-dependent emission, which may affect the air inside the beehive. The variability of this gas results also from the changing weather conditions, especially fluctuations of atmospheric temperature, pressure, and humidity as well as rain and wind. Internal factors include the activity and behavior of honey bees, e.g., changes in foraging activity. Each of those temporal disturbances induces specific variation of sensor signals. Figure 6 presents the results of monitoring of two bee colonies, for one colony in Figure 6a,b, and for the second colony in Figure 6c,d. They inhabited the adjacent beehives of the same kind, in one apiary. Measurements were carried out on two days. The results collected on day I are shown in Figure 6a,c and the results collected on day II are shown in Figure 6b,d. Clearly, the air in the beehives of two bee colonies displayed different characteristics and temporal variability patterns, although the ambient conditions in the monitoring period were the same. The factors associated with the colony itself dominated the information contained in gas sensing device readings. It should be underlined that the observed temporal variation of sensor signals contains relevant pieces of information, and it should not be confused with noise, which is random and erratic.

The changes of beehive air very often present specific patterns of occurrence. The most obvious cycles of variation are seasonal and diurnal. The seasonal cycle is associated with the meteorological conditions which exhibit quite a predictable annual pattern of changes. The diurnal cycle of variation is mostly caused by a daily pattern of bee activity. As beehive air characteristics may vary in different time scales, the selection of the appropriate time for measurements and the duration of the measurement session is a particularly important issue.

In other words, there has to be a chosen number of *‘exposure-cleaning’* operations that should be carried out. If the level of temporal variability of beehive air is high, a larger number of *‘exposure-cleaning’* operations need to be performed. Many *‘exposure-cleaning’* operations give a chance to obtain the results, with better precision and accuracy. However, long measurement sessions require time and resources. The universal solution of this problem is unknown. Only the detailed observation of the environmental factors and understanding of honey bee colony biology can give a clue to whether the duration of measurement session is sufficient to obtain the appropriate data.

The important issue is also the time of the day when the measuring sessions should be performed. It was demonstrated that the adequate time of sampling and measurements allows to obtain better effectiveness of bee disease detection [26]. This knowledge is important from an economical point of view because it helps in planning an effective sampling program for the whole apiary.

### 4.2. Sampling System

The dynamic sampling is performed by a suitable pneumatic system. In this work, the role of the sampling system was to withdraw the representative portion of beehive air and convey it to the sensor chamber. Our sampling system was easily adaptable to different field conditions thanks to modular construction. It consisted of a sample probe, filtration unit and the sample delivery system. The construction of a sampling system is largely dependent on the type of gas to be tested. Physical adsorption on solid surfaces, chemical activity and stability of various beehive air components are different. Therefore, materials used for the sampling operations and construction of the sampling equipment that come into contact with the beehive air must be such that they do not significantly affect the composition of gas sample.

#### 4.2.1. Sample Probe

The insertion of a sample probe in the hive should be to a maximum non-intrusive, in order to avoid modifying the bees’ environment. In addition, the sampling procedure and the probe material cannot change the chemical composition of a gas sample.

**Figure 7:**
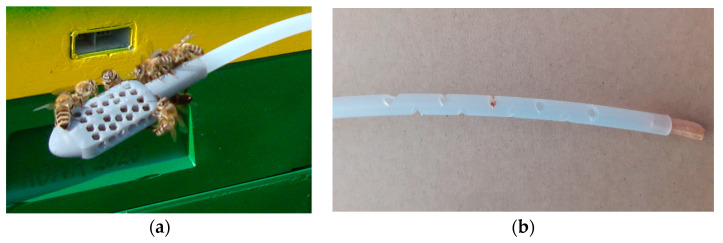
Sample probes used in our studies: (**a**) probe made in advance by 3D printing; the thickness of the probe is greater than the gas line diameter (6/4 mm); (**b**) probe invented and made in field conditions. The gas line was cut with small holes, close to its end. The probe has the diameter of the gas line.

Two types of sampling probes were tested in our studies. These probes are shown in Figure 7. The first one was prepared in advance by a 3D printing technique. The first kind of probe was very convenient, provided that the probe could be located between combs and placed there by accessing the beehive from the top. There was not an observed probe interference with comb maintenance by the bees. The second probe was invented in the apiary, in response to the urgent need. We found that a beekeeper may not agree to disturb bees by opening the beehive for probe installation. In such a case, the air has to be sampled via the exit of the hive. However, in a moderate climate, late summer, beehive exits are narrowed to prevent robbing by pests, e.g., mice. Gas sampling through the beehive exit requires that the sample probe has a small diameter, it is relatively stiff (not to move around the bottom of the beehive) and allows for collecting air over a certain distance, rather than from nearly one point. This is because one does not see the actual location of the probe when accessing the beehive this way. This goal was largely attained by the second kind of probe. It was made by cutting the gas line (PE) with small cuts, close to its end, at the distance of about 10 cm. We did not notice that the construction of the probe was reflected in the measured sensor signals, i.e., readily changed their character. However, it should be mentioned that bees tend to cover foreign objects inside the hive with propolis or wax, which would interfere with the beehive air access to the sampling probe and its movement across the probe. So, this element must be checked with some regularity to ensure that it is sufficiently clean. The first construction was more prone to clogging.

#### 4.2.2. Sampling Point Selection

**Figure 8:**
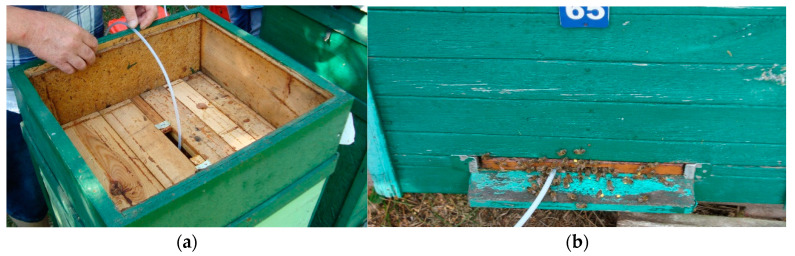
Different location of the sampling point inside the beehive: (**a**) between combs and (**b**) under combs, at the bottom of the beehive.

The sampling probe location in the measured object is of the utmost importance for the measurement process. In a closed system, where the gas composition is homogeneous and unchanged, samples may be taken anywhere within the object. However, the composition of beehive air varies within a beehive [27]. Hence, the selection of an appropriate sampling site was of paramount importance in our study. The spatial variability of beehive air results from the construction of a beehive, behavior of honey bee colony and the influence of the outdoor environment. Additionally, the construction of the beehive as well as the attitude of the beekeeper has a strong influence on the possibility to install the sample probe in a particular location, for example, between combs, see Figure 8a, or at the bottom of the beehive, see Figure 8b.

**Figure 9:**
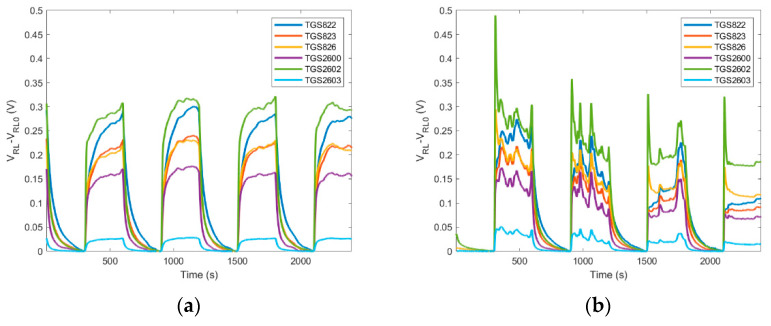
Gas sensor responses to beehive air when the probe was located: (**a**) between combs, (**b**) near the beehive exit.

Based on our measurements, the differences among sensor responses to gas sampled at various points within a hive could be large. For example, the placement of the probe near the beehive exit usually resulted in strongly fluctuating sensor signals. The exemplary measurement results collected in such circumstances are shown in Figure 9b. Contrarily, when the air was sampled from the space between the combs, sensor signals displayed less variability. The respective example of measurement results is shown in Figure 9a.

In our opinion, the placement of the sampling probe should be standardized, at a fixed location with respect to the hive, such as, e.g., at the middle frame in the brood box, or with respect to the colony, such as, e.g., the center of the brood cluster. Theoretically, such an approach allows to perform sampling at very precise locations within the hive. In practice, it was observed that the location of the sampling probe changed over time. It was caused directly by the bees’ movement, and indirectly, by the effects of their activity, e.g., the changing size of the brood clusters. Multi-point sampling at selected points was considered, but in our opinion, it was too invasive.

#### 4.2.3. Sample Conditioning Unit

Interferences represent one of the key factors influencing measuring characteristics of gas sensing devices. They may result from numerous sources. The identification and quantification of complex gas mixtures is negatively affected in the presence of chemical interferences above the expected levels. Sometimes, the sample conditioning system designed for the removal of interfering substances helps to reduce this problem. For the application in question, we considered the incorporation of filtration and drying of the sampled gas.

Beehive air contains gases as well as particulate matter. Particulate filters, installed in holders, were used to extract the solid particles from the sampled gas. Glass fiber, polytetrafluoroethylene (PTFE) and nylon filters were tested. Finally, PTFE filters with a high retention rate and long service life were applied in the sampling system, inside the instrument—see Figure 3. It was assumed that their replacement would be carried out by a qualified technical service, once per season. Additionally, syringe nylon filters 25 mm, 0.45µm were applied. They were mounted externally on the sample gas line, between the beehive and the measurement device and at the end of air sampling line—see Figure 1. They provided the first step protection of sensors against the solid particles contained in the beehive and atmospheric air. These filters were recommended for replacement after one day of measurements. The exchange was easy, so filter cleaning was not practiced.

**Figure 10:**
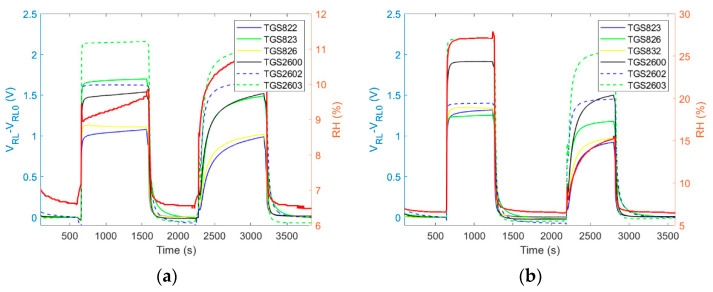
(**a**) Gas sensor responses to dried air, which contained ethanol vapors when: (left) sample was transferred directly to the measuring device and (right) and sample passed the Nafion dryer prior to measurement. (**b**) Gas sensor responses to humid air, which contained ethanol vapors when: (left) sample was transferred directly to the measuring device and (right) and sample passed the Nafion dryer prior to measurement.

In gas sensor measurements, the most common interference results from water vapor. The sensor sensitivity to this compound is a very serious weakness in an electronic nose technology because the signals generated in the semiconductor as sensors are strongly affected by gas humidity changes. In our studies, the permeation dryer based on a bundle of Nafion tubes was tested in view of applying it for beehive air sample conditioning. Nafion is a copolymer of tetrafluoroethylene (Teflon) and perfluoro-**3**,**6**-dioxa-**4**-methyl-**7**-octene-sulphonic acid. This material is highly selective in the removal of water, while most of other substances are retained quantitatively. However, although they do not attack or damage the Nafion tubing, some polar organic substances are absorbed by it and consequently lost from the gas sample. The integrity of the sample is not preserved. Figure 10 presents the results of the laboratory experiment, where we tested the gas sensor responses to dry and humid air containing ethanol vapors, as an example of polar VOCs. The gas sensor response to dried air containing ethanol decreased, when the sample passed the Nafion dryer prior to sensor measurement (Figure 10a). Clearly, some ethanol was removed from the gas sample due to this operation. Similar observation was made for humid air containing ethanol vapors. Sensor responses were changed when gas sample passed Nafion dryer prior to measurement (Figure 10b), while relative humidity reduction was limited. Based on the experiments, we concluded that the application of Nafion dryers for beehive air sample conditioning is limited.

In general, the elimination of chemical interferences is one of the greatest challenges in practical applications of gas sensing devices. This problem has not been solved yet. The sample conditioning systems can have a negative influence on the representativeness of beehive air measurements. In our approach, interferences resulting from water vapor were taken into account at the level of the measurement data processing.

#### 4.2.4. System of Gas Delivery

The system of gas delivery was designed to transport a volume of the beehive air from the sampling point to the measurement chamber and to maintain the gas composition throughout the sampling system for the sensor measurement. It included a gas line, valves to control the gas flow and gas mover (a pump).

**Figure 11:**
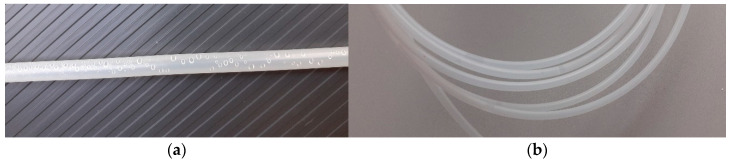
Condensate, which was formed inside gas line, during beehive air measurements: (**a**) initially condensate adheres to the walls of the tubing, (**b**) later, the amount of condensate may be sufficient to block the tubing.

The gas delivery system cannot cause degradation and loss of the sampled gas prior to measurements. The chemical complexity of beehive air must be maintained. The appropriate tubing can reduce the chance of sample decomposition. Sample lines must be made of inert material. Polytetrafluoroethylene (PTFE) was selected as a tubing material inside the sensor device. Outside, polyethylene tubing was used. Adsorption of beehive air components on the surface of this material was low. However, oftentimes, condensate was formed on the inner wall of the tubes. The water condensation played the main role in this process. During sampling, the gas sample inside the tube is under the influence of ambient conditions. Physical conditions inside and outside the beehive are usually different. The brood nest temperature is of extreme importance to the colony, and it is controlled with the utmost precision. Honey bees maintain the temperature of the brood nest between 32 °C and optimally 35 °C. The relative humidity inside the beehive is not constant. The bees try to maintain an average humidity value of 60% inside. The maximum amount of water vapor that can be present in beehive air before it condenses (reach the saturation point) depends almost entirely upon the outside temperature. The water condensate was formed in the delivery system, when the temperature of the tube walls was lower than the dew point of the gas sample. Such an effect was observed during our measurement sessions many times, as shown in Figure 11.

**Figure 12:**
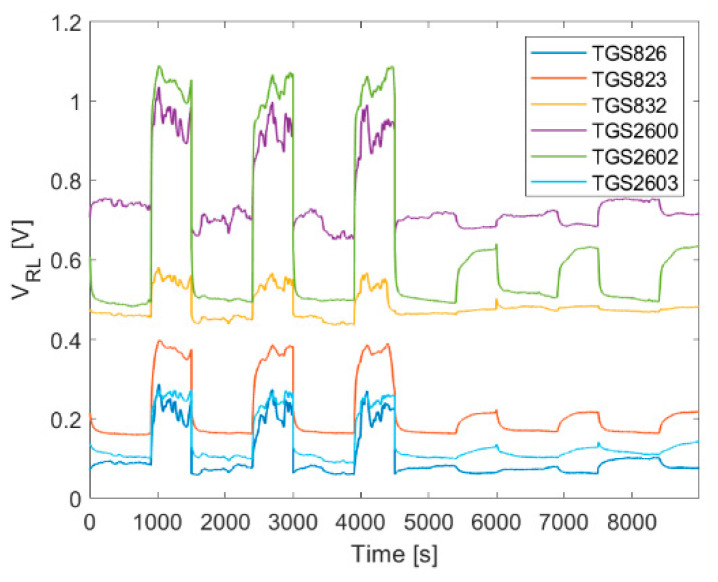
Sensor signals change due to wetting of the first step filter. Wetting took place after the third test measurement.

The water condensation has an adverse impact on the measuring equipment and must be eliminated. If it occurs, there is a risk that the first step filter will be completely wettened. In turn, the first step filter wetting is reflected in gas sensor signals. The exemplary measurement results, which illustrate such a situation, are shown in Figure 12. The first three sets of sensor signals were recorded when the first step filter was dry. Due to filter wetting, signals suddenly decreased, and they remained at the new low level. In such a case, the composition of the measured gas sample does not reflect the beehive air composition. The sample is not representative and the measurement results are erroneous.

Water vapor condensation poses a risk of the first step filter clogging. When unnoticed, it may lead to the damage of the electronic elements of the sensor device, and also the pump.

Transfer lines may be heated to prevent water condensation during sample transport when a temperature drops below the dew point. We developed a heated sample line in this study. It consists of PTFE tubing surrounded by the heating coil and insulation with an outer protective sleeve. A spiral-wound heating element for uniform heat distribution was used. Silicone or elastomer foam was used as the thermal isolation. Electricity for the heated sample line was supplied from batteries, which power the sensor device. The power consumption of the heated gas line was 7 W, which is high compared with the power consumption of the gas sensing device without it (see Section 3.1). The heated sample lines should be lightweight and flexible. In practice, it is difficult to fulfil this requirement. Our heated sample line caused logistic problems.

The problem of water condensation could be partially solved by adjusting the length of the sample line. It must be as short as possible, with no bends between the sampling point and the gas sensing device. The short residence time of the tested gas in the sampling system is very important. The sampling system in our work had a sample residence time less than 1.5 s. However, water condensation was sometimes observed. Therefore, inspection of the gas line by the beekeeper is recommended.

Dynamic sampling is liable for leakage. However, for assuring gas sample integrity, it is not allowed that the tested gas becomes diluted by the atmospheric air during transfer to the sensor chamber. Therefore, the appropriate construction and gas-tightness of the sampling system is a principal requirement. In field conditions, connectors and adapters play an especially important role. Quick fastening gas-tight connections were successfully used. The sampling system must be regularly examined and tested for leaks.

Pumps are required in order to convey the sample to the sensor chamber. In our gas sensor device, we used the miniature eccentric diaphragm pump for gases SP 570 EC. Pulse-width modulation (PWM) was used for pump performance control. The beehive air was automatically aspirated by the pump at a prescribed flow rate and sampling time. The pump was located after the measuring unit. The pump had the capability to extract the gas from the beehive as well as to draw a reference air for cleaning operation. The flow capacity of the pump should be large enough to prevent long residence time of gas sample inside the measurement system. It has to be airtight because the beehive air cannot be diluted. Dynamic sampling requires power. In most realistic measuring systems, there is a limit of power supply. Thus, the power consumption of the pump has to be relatively low (see Section 3.1).

It was not observed that the gas sensing device operation in field conditions imposed the need for frequent replacement of the sampling system components, except for the first step filters. However, proper maintenance of all sampling equipment is vital to the proper operation of the measuring device. The sample probe and gas lines must not be clogged or somehow obstructed. They should be kept clean and dry, free of water condensate.

## 5. Conclusions

This paper discussed the selected issues regarding the application of gas sensing devices in beekeeping. They are beehive air sampling and the operation of the instrument, which is subordinate to the sampling method. Our attention was mainly focused on the impact of the environmental factors (beehive and ambient environments), as they significantly affect measurement results and gas sensing device operation in field conditions. The main conclusions are as follows:

- Dynamic sampling allows for the collection of representative samples of beehive air, and its unobstructed delivery to sensors chamber, which results in high accuracy of measurement. Proper gas flow rate selection is crucial.
- The ‘*exposure-cleaning*’ operation allows for performing short-term measurements as well as long-term monitoring of beehive air. It prevents measurement bias and assures good temporal resolution of the acquired information. An adequate duration of exposure and cleaning operations is important.
- The sampling system cannot change the composition of the gas sample. Proper construction materials are needed for this purpose; we chose PTFE and PE.
- The sample probe geometry has to be defined on the basis of the intended localization of the sampling point in the beehive. Assess constraints apply.
- The location of the sampling point in the beehive should be strictly defined, as it influences gas sensor responses.
- The sampling system has to be fitted with elements that protect the sensing device from particulate matter and water condensate. This role is fulfilled by adequate filters. A Nafion drier was excluded as it changed the chemical composition of the gas sample.
- Elements of the sampling system have to be checked, cleaned, dried, or replaced as a part of regular daily maintenance of the gas sensing device. It is necessary for attaining proper measurement results and to prevent the damage of the instrument.

We believe that the information presented in this paper is valuable for designers and constructors of gas sensing devices. We draw attention to the problems that have a general character, while their solutions are heavily dependent on the kind of application. We hope that sharing our knowledge and experience will contribute to progress in developing gas sensing devices for apiculture.

## Data Availability

Data sharing is subject to restrictions.

## References

[1] Gallai N., Salles J.M., Settele J., Vaissière B.E. (2009). Economic valuation of the vulnerability of world agriculture confronted with pollinator decline. Ecol. Econ..

[2] Watson K., Stallins J.A. (2016). Honey Bees and Colony Collapse Disorder: A Pluralistic Reframing. Geogr. Compass.

[3] Sperandio G., Simonetto A., Carnesecchi E., Costa C., Hatjina F., Tosig S., Gilioli G. (2019). Beekeeping and honey bee colony health: A review and conceptualization of beekeeping management practices implemented in Europe. Sci. Total Environ..

[4] Zacepins A., Stalidzans E., Meitalovs J. Application of information technologies in precision apiculture. Proceedings of the 13th International Conference on Precision Agriculture (ICPA 2012).

[5] Bozek K., Hebert L., Portugal Y., Stephens G.J. (2021). Markerless tracking of an entire honey bee colony. Nat. Commun..

[6] Ferrari S., Silva M., Guarino M., Berckmans D. (2008). Monitoring of swarming sounds in bee hives for early detection of the swarming period. Comput. Electron. Agric..

[7] Sánchez V., Gil S., Flores J.M., Quiles F.J., Ortiz M.A., Luna J.J. (2015). Implementation of an electronic system to monitor the thermoregulatory capacity of honeybee colonies in hives with open-screened bottom boards. Comput. Electron. Agric..

[8] Meikle W.G., Weiss M., Maes P.W., Fitz W., Snyder L.A., Sheehan T., Mott B.M., Anderson K.E. (2017). Internal hive temperature as a means of monitoring honey bee colony health in a migratory beekeeping operation before and during winter. Apidologie.

[9] Bilik S., Kratochvila L., Ligocki A., Bostik O., Zemcik T., Hybl M., Horak K., Zalud L. (2021). Visual Diagnosis of the Varroa Destructor Parasitic Mite in Honeybees Using Object Detector Techniques. Sensors.

[10] Arenas A., Fernandez V.M., Farina W.M. (2008). Floral scents experienced within the colony affect long-term foraging preferences in honeybees. Apidologie.

[11] Mas F., Horner R., Brierley S., Harper A., Suckling D.M. (2020). The Scent of Individual Foraging Bees. J. Chem. Ecol..

[12] Edwards-Murphy F., Magno M., Whelan P.M., O’Halloran J., Popovici E.M. (2016). b+WSN: Smart beehive with preliminary decision tree analysis for agriculture and honey bee health monitoring. Comput. Electron. Agric..

[13] El-Wahed A.A.A., Farag M.A., Eraqi W.A., Mersal G.A.M., Zhao C., Khalifa S.A.M., El-Seedi H.R. (2021). Unravelling the beehive air volatiles profile as analysed via solid-phase microextraction (SPME) and chemometrics. J. King Saud Univ. Sci..

[14] Cecchi S., Terenzi A., Orcioni S., Spinsante S., MarianiPrimiani V., Moglie F., Ruschioni S., Mattei C., Riolo P., Isidoro N. (2019). Multi-sensor platform for real time measurements of honey bee hive parameters. IOP Conf. Series Earth Environ. Sci..

[15] Meikle W.G., Holst N. (2015). Application of continuous monitoring of honeybee colonies. Apidologie.

[16] Cheng L., Meng Q.-H., Lilienthal A.J., Qi P.F. (2021). Development of compact electronic noses: A review. Meas. Sci. Technol..

[17] Szczurek A., Maciejewska M., Bąk B., Wilk J., Wilde J., Siuda M. (2020). Gas sensor array and classifiers as a means of varroosis detection. Sensors.

[18] Szczurek A., Maciejewska M., Bąk B., Wilk J., Wilde J., Siuda M. (2020). Detecting varroosis using a gas sensor system as a way to face the environmental threat. Sci. Total Environ..

[19] Borecki M., Duk M., Kociubiński A., Korwin-Pawlowski M.L. Multiparametric Methane Sensor for Environmental Monitoring. Proceedings of the SPIE 10175, Electron Technology Conference 2016.

[20] Miskell G., Salmond J., Grange S., Weissert L., Henshaw G., Williams D. (2017). Reliable Long-Term Data from Low-Cost Gas Sensor Networks in the Environment. Proceedings.

[21] Zhang Y., Szabo C., Sheng Q.Z., Benatallah B., Bestavros A., Manolopoulos Y., Vakali A., Zhang Y. (2014). Cleaning Environmental Sensing Data Streams Based on Individual Sensor Reliability. Lecture Notes in Computer Science, Proceedings of the Web Information Systems Engineering—WISE 2014, Thessaloniki, Greece, 12–14 October 2014.

[22] Betty C.A., Choudhury S., Girija K.G. (2014). Reliability studies of highly sensitive and specific multi-gas sensor based on nanocrystalline SnO_2_ film. Sens. Actuators B Chem..

[23] Szczurek A., Maciejewska M. (2013). Gas sensing method applicable to real conditions. Meas. Sci. Technol..

[24] Szczurek A., Maciejewska M., Zelek M. (2014). Influence of gas sampling on MOS response in real measurement conditions. Procedia Eng..

[25] Figaro Sensor. https://www.figaro.co.jp/en/product/sensor/.

[26] Szczurek A., Maciejewska M., Zajiczek Ż., Bąk B., Wilk J., Wilde J., Siuda M. (2020). The effectiveness of Varroa destructor infestation classification using an E-nose depending on the time of day. Sensors.

[27] Szczurek A., Maciejewska M., Bąk B., Wilk J., Wilde J., Siuda M. (2020). Beehive indoor air measurement for bee colony diagnostics. Indoor Air 2020: Creative & Smart Solutions for Better Built Environments, Proceedings of the 16th Conference of the International Society of Indoor Air Quality & Climate, Seoul, Korea, 1 November 2020.

